# A systematical procedure to extracting legal entities from Indonesian judicial decisions

**DOI:** 10.1016/j.mex.2025.103610

**Published:** 2025-09-06

**Authors:** Eka Qadri Nuranti, Naili Suri Intizhami, Evi Yulianti, A. Muh. Iqbal Latief, Osama Iyad Al Ghozy

**Affiliations:** a Department of Computer Science, Institut Teknologi Bacharuddin Jusuf Habibie, Parepare, Indonesia; b Department of Computer Science, Faculty of Computer Science, Universitas Indonesia, Depok, Indonesia; c Kejaksaan Negeri Wajo, Indonesia

**Keywords:** Information Extraction, Text processing, Name entity recognition, Legal document, Dataset

## Abstract

This article presents a systematic method of extracting legal entities from Indonesian judicial decisions with a well-structured named entity recognition (NER) approach. The procedure was implemented by gathering and annotating court decisions for theft cases at three court levels: first instance (2478 files), appeal (147 files), and cassation (62 files), amounting to 2687 annotated files. The data were harvested from the official website of the Supreme Court of the Republic of Indonesia using automated web scraping, followed by manual filtering for relevance and completeness.

Manual annotation was performed with the Label Studio platform by three independent annotators. Annotation consistency was considered using Fleiss' Kappa, yielding an average agreement score of 0.705 across all levels, indicating good inter-annotator reliability. The method uses a hierarchical structure and a BIO tagging scheme to tag >50 types of legal entities, including defendants, judges, legal articles, charges, and verdict decisions.

This approach is proper for text processes such as legal information extraction, classification, and legal analysis. From a legal perspective, this process will improve legal transparency and research on Indonesian judicial data.

• Structured pipeline for gathering, filtering, and annotating Indonesian court judgments based on legal metadata and web scraping.
• Manual annotation of 2687 court documents with annotation rules and inter-annotator agreement using Fleiss' Kappa.
• Token-level translation and BIO tagging for >50 legal entities, enabling downstream NLP tasks such as named entity recognition.

## Graphical abstract

**Figure:**
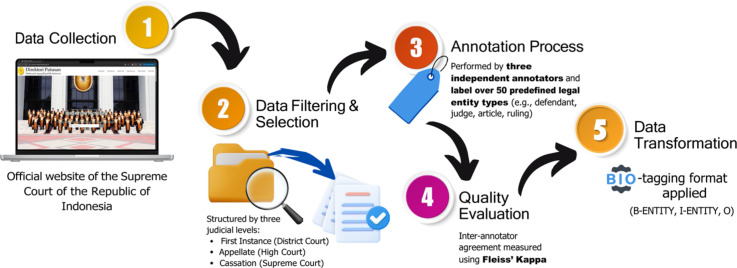

## Background

The method outlined in this article aims to bridge a gap of particular concern in legal Natural Language Processing by developing a reproducible pipeline to retrieve legal entities from Indonesian court decisions. With legal documents increasingly accessible via the official website of the Supreme Court of the Republic of Indonesia, researchers can utilize rich, real-world data to reflect judicial processes. This open access is a significant step towards facilitating legal transparency and enabling computational legal analysis.

While earlier research has initiated legal NLP for Indonesian documents, most activities have been on limited domains. For example, Solihin et al. [1] introduced an annotated dataset of 150 theft case decisions to examine rule-based entity recognition. Nuranti et al. [2] continued this research with a more considerable corpus of 1003 general criminal judgments and applied neural models such as BiLSTM+CRF. Yulianti et al. [3] applied large language models (LLMs) such as XLM-R and IndoRoBERTa on 993 labeled decisions in more recent research, focusing on verdict-related entity recognition.

These works have set foundations for Indonesian legal NLP but tend to deal with one-level cases (usually first instance) and are yet to be comprehensive across the judicial hierarchy. As pointed out by Solihin et al. [4], no detailed and standardized annotated corpora can be used as a benchmark for legal information extraction.

To address these constraints, the approach devised in this study establishes an end-to-end pipeline spanning multiple judicial tiers—initial trial, appellate review, and cassation—and incorporates elements such as manual annotation, inter-annotator consistency evaluation, and strict transformation of outputs. The approach targets theft cases to ensure thematic consistency while incorporating a hierarchical judicial system within the annotation pipeline. The goal is to create an annotated corpus and provide a reproducible and scalable approach to curating and preparing legal decision text for NER tasks. The approach facilitates the structured extraction of legal entities like defendants, judges, legal articles, and verdicts, allowing one to train solid NLP models for legal information extraction. This methodological approach can be reused, modified, and applied to other case types or jurisdictions, thus assisting in a broader endeavor to construct AI-ready legal corpora for low-resource linguistic settings such as Bahasa Indonesia.

### Method details

This method establishes a systematic process of legal entity extraction from Indonesian court decision documents employing structured manual token-level tagging and annotation. The process allows for enhancing and testing NER models in the legal field, particularly for less-resourced languages such as *Bahasa Indonesia*.

The approach was crafted to be replicable, scalable, and flexible across various legal domains and jurisdictions. It entails five key steps:

**1. Data Collection**

The data utilized in this study are court judgment documents that were gathered using a web scraping technique in Python from the official website of the Supreme Court of the Republic of Indonesia. The general criminal cases are the primary concern, with particular attention to the theft (Indonesian: *pencurian*) cases, which have been selected to reveal the complexity of the legal system in three stages: First Instance, Appeal, and Cassation.

Every case that had been gathered from the website contained two general parts:

• The court's full judgment document is usually filed in PDF format.
• A formal metadata entry linked to each decision includes the following fields: a unique identifier, court level (First Instance, Appeal, Cassation), *Inckrah* (if the case has attained legal finality), case classification (e.g., Theft), and textual domains that articulate the judicial decision.

**2. Data Filtering and Selection**

To provide legal completeness and relevance, every document collected underwent a manual screening process reliant on the metadata and content of each document. The documents were reviewed to verify the existence of essential legal elements, such as the complete court ruling, legal reasoning, and final judgment. Incomplete documents, which were characterized by missing important sections and showing corrupted formatting, were excluded from the annotation process.

Implementing this filtering process was critical to upholding the integrity of the dataset, such that only documents of legal significance and meaningful content were selected for manual annotation. The dataset is, therefore, representative, consistent, and appropriate for judicial decisions for downstream natural language processing tasks.

In addition to manual review, Case metadata served a central role in the filtering process, allowing for identifying and selecting reports included in criminal cases involving Theft and had been wholly adjudicated (i.e., *Inckrah* marked complete).

**Fig. 1:**
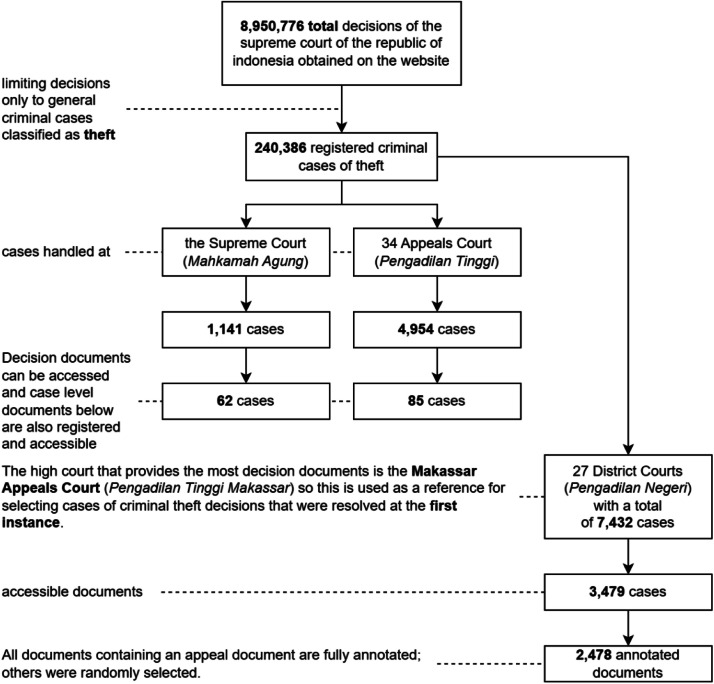
Distribution of documents of court decisions in general criminal cases of theft.

Moreover, Fig. 1 outlines the systematic document retrieval process using the Prisma method, emphasizing the structured approach to collecting data from the Supreme Court's website. The figure also shows the distribution of theft cases across the judicial levels, the number of documents annotated manually, and the plan for automatic annotation of the remaining documents.

This research has collected 3479 theft cases with court decision documents available for analysis. These cases are distributed across the three judicial levels as follows:

• First Instance: 3479 documents were available, but only 2487 were manually annotated.
• Appellate: 147 documents at the appellate level were obtained from 85 appeal cases and 62 cases related to cassation cases.
• Cassation: 62 cases with available documents at the cassation level.

**3. Annotation Process**

All entity labels are normalized and expressed in English to ensure international applicability and consistency in this framework. The annotation schema was developed collaboratively and then encoded in extensive annotation guidelines. The guidelines were used as a basis for training annotators and for defining boundaries and characteristics for every category of entities. The entity schema covering information such as: Parties and roles: Defendant, Lawyer, Prosecutor, Witness, PresidingJudge; Legal process: ProsecutionDate, DecisionDate, ArrestDate, CassationReason; Legal references: ChargeArticles, ProsecutionArticles, CourtRuling, DecisionCosts; Case identifiers and metadata: DecisionNumber, ChargeType, CaseLevel, IncidentLocation. Some entities are only available at certain levels, such as the grounds for cassation found in the Supreme Court. The annotations for this dataset provide a complete list of entities, a brief description, and the level of justice explicitly detailed in Table 1.

**Table 1:** List of annotated entities.

No	Entity Name	Entity Description	Level of Justice		
			First Instance	Appellate	Cassation
1	AdditionalCharges	Additional accusations filed against the defendant.	✓		
2	AggravatingFactors	Circumstances that may lead to a harsher sentence.	✓		
3	AppellateContent	The written decision issued by the appellate court.		✓	
4	AppellateCosts	Monetary penalties determined at the appellate level.			✓
5	AppellateDecisionDate	The date when the appellate court issued its decision.			✓
6	AppellateNumber	Unique identification for appellate court decisions.			✓
7	AppellateRulingStatement	The appellate ruling referenced in cassation cases.			✓
8	ArrestDate	The date on which law enforcement detained the defendant.	✓	✓	✓
9	AssociateJudge	Judges who assist the presiding judge in the case.	✓	✓	✓
10	CaseLevel	The level of the judiciary handling the case (First Instance, Appellate, Cassation).	✓	✓	✓
11	CaseType	The classification of legal disputes (e.g., criminal, civil).	✓		
12	CassationApplicantReason	The justification provided by either the defendant or prosecutor for filing a cassation.			✓
13	CassationReasonDef	Legal justification presented by the defendant in the cassation stage.			✓
14	CassationReasonPU	Legal justification presented by the prosecutor in the cassation stage.			✓
15	ChargeArticles	The legal provisions referenced in the prosecution’s charges.	✓	✓	✓
16	ChargeDate	The date on which formal charges were filed against the defendant.		✓	
17	ChargeLabel	A label used to classify the charges filed against the defendant.	✓	✓	✓
18	ChargeNumber	The official identification number for the charges filed.		✓	
19	ChargeType	The categorization of the defendant’s offenses.	✓	✓	✓
20	CourtClerk	The official responsible for managing court documents.	✓	✓	✓
21	CourtInstitution	The court responsible for hearing and deciding the case.	✓	✓	✓
22	CourtRuling	Official decision issued by the court regarding a case.	✓	✓	
23	DecisionCosts	The amount the defendant is required to pay based on the court ruling.	✓	✓	✓
24	DecisionDate	The official date when the court issued its decision.	✓	✓	✓
25	DecisionNumber	A unique reference number for the final court ruling.	✓	✓	✓
26	Defendant	The person being prosecuted in the case.	✓	✓	✓
27	DefendantBirthDate	The specific birth date of the defendant, as recorded in legal documents.	✓	✓	✓
28	DefendantDoc	Legal documents provided by the defendant or their counsel.			✓
29	DefendantOccupation	The profession or work status of the defendant.	✓	✓	✓
30	DefendantResponse	The defendant’s response to the prosecutor’s claims.	✓		
31	DefenseCharges	The charges raised by the defendant’s legal counsel during the trial.	✓		
32	DefenseContent	The primary arguments presented in the defendant’s plea.	✓		
33	DefenseResponse	The prosecutor’s counter to the defendant’s plea.	✓		
34	FirstCosts	Monetary penalties set at the first-instance court level.		✓	✓
35	FirstEvidence	Evidence included in the ruling at the first-instance court level.		✓	✓
36	FirstRuling	The ruling issued at the first-instance (district) court.		✓	✓
37	FirstRulingStatement	The first-instance ruling referenced in appellate or cassation cases.		✓	✓
38	FirstRulingUpheld	Confirmation that the first-instance decision was upheld in the appellate court.		✓	
39	IncidentDate	The date when the alleged crime or event occurred.	✓	✓	✓
40	IncidentLocation	The geographical location where the incident related to the case took place.	✓	✓	✓
41	Lawyer	The attorney providing legal representation for the accused.	✓	✓	
42	LegalConsiderations	Legal articles used as references in the judge’s decision-making.	✓	✓	✓
43	LegalOfficeAddr	The official address of the legal counsel representing the defendant.		✓	
44	MitigatingFactors	Conditions that may lead to a lighter sentence.	✓		
45	PresidingJudge	The main judge responsible for overseeing the court proceedings.	✓	✓	✓
46	ProsecutionArticles	The legal articles used as the basis for the prosecution's case.	✓	✓	✓
47	ProsecutionCosts	Financial costs requested by the prosecutor during the case.	✓	✓	✓
48	ProsecutionDate	The date when the prosecutor officially submitted the case.		✓	✓
49	ProsecutionEvidence	Items presented by the prosecutor as evidence in the case.	✓	✓	✓
50	ProsecutionNumber	The number assigned to a case handled by the prosecutor.		✓	
51	ProsecutionSentence	The penalty recommended by the prosecutor.	✓	✓	✓
52	Prosecutor	The legal officer responsible for prosecuting cases in court.	✓		
53	ProsecutorDoc	Legal documents submitted as part of the prosecution’s case.			✓
54	RulingEvidence	Any evidence referenced in the court's final decision.	✓		
55	RulingStatement	The main ruling statement mentioned in the document.	✓	✓	✓
56	Witness	A person who provides testimony in court.	✓		

The data annotation process is crucial in this study to produce a high-quality dataset ready for use in the NER task on court documents. This stage begins with determining an annotation scheme between case levels, designed based on important elements in the decision document to represent relevant legal information. Information about the legal entity agreed upon in this study is based on Table 1.

Three annotators were selected to carry out the annotation process on this dataset. Before starting their task, the annotators underwent intensive training covering two main aspects. First, they participated in preparing an annotation guide to ensure that entity categories and annotation procedures were clearly and consistently defined. Second, they were given training in the use of annotation software, namely Label Studio [5], which is used to mark entities in documents efficiently.

A case study was conducted using document samples from each level of justice to establish a consistent understanding among the annotators. A total of 30 document samples were taken from each level (first instance, appeal, and cassation) to be tested on the annotators. This process helps identify potential misunderstandings, refine the annotation guidelines, and ensure all annotators are on the same page before annotating the entire dataset. These steps are designed to improve the annotation quality and ensure that the resulting dataset is highly accurate and ready for further research.

Once training is complete and annotation guidelines are agreed upon, the annotation process is carried out step by step on each document in the dataset. Three independent annotators manually annotate each document to ensure data quality and consistency. The process consists of several steps: Annotators read the document thoroughly to understand the relevant legal context and structure. Second step: Annotators use Label Studio software to tag text based on pre-defined entity categories. Each entity is given a specific label according to the annotation guidelines, including the start and end positions of the text (start-end indices) in the document. After completing the annotation on a document, annotators review it again to check for any missed entities or errors in tagging. This process is done iteratively to ensure that all documents in the dataset are annotated according to the agreed guidelines.

Tables 2, 3, and 4 present detailed statistics on the entities annotated in court decision documents at three levels of the judiciary: First Instance, Appellate, and Cassation. These statistics include the number of documents containing each entity, the total occurrence of the entity, the number of words contained in the entity, and the average number of words per occurrence of the entity. These data provide an overview of the distribution of entities in judicial documents at each level, which can be used for further analysis and NLP model development.Manual annotation of legal NLP tasks, especially in court documents, requires diligent attention to structure, terminology, and consistency. Due to the hierarchical structure of the Indonesian court system and the variation of information between first-instance, appellate, and cassation decisions, a systematic and standardized annotation process was necessary. This standardization allows the extracted entities to remain consistent and comparable between judicial levels, providing a solid basis for downstream analysis and model training.

**Table 2:** The statistics of entities annotated in first instance court decision documents.

No	Entity Name	Number of Documents	Number of Occurrences	Number of Words	Average Words per Entity
1	AdditionalCharges	11	32	397	12.406
2	AggravatingFactors	2445	4319	28,633	6.63
3	ArrestDate	1038	1075	3335	3.102
4	AssociateJudge	2462	4992	28,203	5.65
5	CaseLevel	2463	2464	4936	2.003
6	CaseType	2468	2471	5676	2.297
7	ChargeArticles	2313	3185	29,254	9.185
8	ChargeLabel	815	1673	1814	1.084
9	ChargeType	2387	2402	2580	1.074
10	CourtClerk	2475	2478	10,901	4.399
11	CourtInstitution	2476	2478	7525	3.037
12	CourtRuling	2467	2477	40,995	16.55
13	DecisionCosts	2461	2476	21,745	8.782
14	DecisionDate	2465	2473	7487	3.027
15	DecisionNumber	2470	2478	19,799	7.99
16	Defendant	2475	3053	16,391	5.369
17	DefendantBirthDate	2467	3043	9039	2.97
18	DefendantOccupation	2426	2974	5593	1.881
19	DefendantResponse	1973	1980	8169	4.126
20	DefenseCharges	736	746	1062	1.424
21	DefenseContent	2355	2407	44,022	18.289
22	DefenseResponse	1911	1911	6367	3.332
23	IncidentDate	2422	2742	8712	3.177
24	IncidentLocation	2428	2696	38,528	14.291
25	Lawyer	259	598	2997	5.012
26	LegalConsiderations	2465	2475	28,284	11.428
27	MitigatingFactors	2453	5846	43,086	7.37
28	PresidingJudge	2475	2477	13,937	5.627
29	ProsecutionArticles	2270	2291	22,647	9.885
30	ProsecutionCosts	2430	2436	23,683	9.722
31	ProsecutionEvidence	2320	8684	104,356	12.017
32	ProsecutionSentence	2428	2487	23,813	9.575
33	Prosecutor	2439	2458	11,822	4.81
34	RulingEvidence	2356	8949	107,865	12.053
35	RulingStatement	2436	2508	25,959	10.35
36	Witness	2472	7998	34,207	4.277

**Table 3:** The statistics of entities annotated in appellate court decision documents.

No	Entity Name	Number of Documents	Number of Occurrences	Number of Words	Average Words per Entity
1	AppellateContent	60	246	10,440	42.439
2	ArrestDate	44	47	144	3.064
3	AssociateJudge	147	296	1672	5.649
4	CaseLevel	146	146	301	2.062
5	ChargeArticles	142	211	2131	10.1
6	ChargeDate	99	99	314	3.172
7	ChargeLabel	58	126	151	1.198
8	ChargeNumber	102	102	1449	14.206
9	ChargeType	57	57	74	1.298
10	CourtClerk	147	148	684	4.622
11	CourtInstitution	147	148	501	3.385
12	CourtRuling	147	293	5233	17.86
13	DecisionCosts	143	143	1414	9.888
14	DecisionDate	146	147	469	3.19
15	DecisionNumber	147	147	922	6.272
16	Defendant	147	206	1061	5.15
17	DefendantBirthDate	147	206	614	2.981
18	DefendantOccupation	144	203	411	2.025
19	FirstCosts	143	144	1328	9.222
20	FirstEvidence	133	758	9617	12.687
21	FirstRuling	145	145	717	4.945
22	FirstRulingStatement	144	157	1070	6.815
23	FirstRulingUpheld	135	140	3834	27.386
24	IncidentDate	139	160	1166	7.288
25	IncidentLocation	140	141	2573	18.248
26	Lawyer	45	96	510	5.313
27	LegalConsiderations	140	144	1661	11.535
28	LegalOfficeAddr	40	41	646	15.756
29	PresidingJudge	147	148	843	5.696
30	ProsecutionArticles	143	144	1635	11.354
31	ProsecutionCosts	146	147	1457	9.912
32	ProsecutionDate	115	116	364	3.138
33	ProsecutionEvidence	136	714	8837	12.377
34	ProsecutionNumber	108	108	1537	14.231
35	ProsecutionSentence	145	152	1082	7.118
36	RulingStatement	144	147	3895	26.497

**Table 4:** The statistics of entities annotated in the cassation court decision documents.

No	Entity Name	Number of Documents	Number of Occurrences	Number of Words	Average Words per Entity
1	AppellateCosts	28	28	198	7.071
2	AppellateDecisionDate	45	45	130	2.889
3	AppellateNumber	57	58	366	6.31
4	AppellateRulingStatement	62	123	2020	16.423
5	ArrestDate	32	33	102	3.091
6	AssociateJudge	61	124	787	6.347
7	CaseLevel	61	62	123	1.984
8	CassationApplicantReason	12	61	3931	64.443
9	CassationReasonDef	24	99	6998	70.687
10	CassationReasonPU	40	165	10660	64.606
11	ChargeArticles	54	84	812	9.667
12	ChargeLabel	26	58	63	1.086
13	ChargeType	38	38	48	1.263
14	CourtClerk	61	61	349	5.721
15	CourtInstitution	61	61	114	1.869
16	DecisionCosts	60	90	772	8.578
17	DecisionDate	62	124	365	2.944
18	DecisionNumber	62	124	759	6.121
19	Defendant	62	85	412	4.847
20	DefendantBirthDate	62	85	250	2.941
21	DefendantDoc	28	32	1531	47.844
22	DefendantOccupation	62	85	158	1.859
23	FirstCosts	61	61	414	6.787
24	FirstEvidence	53	309	3873	12.534
25	FirstRuling	61	61	255	4.18
26	FirstRulingStatement	62	68	469	6.897
27	IncidentDate	11	13	59	4.538
28	IncidentLocation	11	11	193	17.545
29	LegalConsiderations	61	61	666	10.918
30	PresidingJudge	62	62	419	6.758
31	ProsecutionArticles	62	63	660	10.476
32	ProsecutionCosts	62	62	415	6.694
33	ProsecutionDate	57	57	163	2.86
34	ProsecutionEvidence	55	282	3480	12.34
35	ProsecutionSentence	62	66	456	6.909
36	ProsecutorDoc	38	47	2461	52.362
37	RulingStatement	62	66	1003	15.197

Annotations were made using Label Studio, which stores the output in JSON. Each JSON file includes document text and a list of entities annotated, along with start and end character offsets, entity labels, and the text annotated. The JSON file was in the intermediate format before converting to token-level BIO-tagged data for NER model training.

**4. Annotation Validation**

After completing the annotation, we compare the results from the three annotators to calculate the agreement level using the Fleiss’ Kappa metric. This metric works well since three annotators independently annotate each document. Fleiss’ Kappa measures agreement among more than two annotators [6]. Measuring the agreement ensures the annotation’s consistency and accuracy. The formula for Fleiss’ Kappa (K) is as follows:

$$K=\frac{P-P_{e}}{1-P_{e}}$$

Definition:

• P: Actual level of agreement among annotators.
• $P_{e}$: Randomly expected level of agreement.

Fleiss’ Kappa values are interpreted based on the following scale:

• < 0.00: No agreement
• 0.00–0.20: Weak agreement
• 0.21–0.40: Fair agreement
• 0.41–0.60: Moderate agreement
• 0.61–0.80: Good agreement
• 0.81–1.00: Excellent agreement

**5. Data Transformation**

Every document that had been annotated was tokenized using a standardized Python script that linked every token to its corresponding entity span from the JSON annotation output. Tokens were then classified using the BIO tagging scheme tag (e.g., B-Entity, I-Entity, O for outside tokens) to mark entity boundaries, a standard approach in sequence labeling tasks for NER [7].

The converted data were saved in three structured CSV files: 1-first-instance.csv, 2-appellate.csv, and 3-cassation.csv, with columns token, label, and document id. In this way, the structuring achieves consistency with popular machine learning libraries and simplicity in training, evaluation, and subsequent analysis of NER models on legal text [2,8].

Together with metadata.csv, the corpus offers a reusable and organized asset for building NLP models for low-resource legal settings. The whole set of annotated and transformed documents has been published on Mendeley Data with the title IDTheftCase-JudgmentCorpus dataset [9], thus enabling transparency and reproducibility for future natural language processing studies in legal domains.

## Method validation

In order to identify the consistency and reliability of the manual annotation process, an inter-annotator agreement was calculated using Fleiss' Kappa. Validation here is necessary to verify if the annotation guidelines were consistently followed by all annotators and across various levels of the judiciary. Agreement scores convey information regarding how good and appropriate the resulting dataset is for subsequent natural language processing steps.

Table 5 presents the results of the Fleiss’ Kappa calculations to measure the level of agreement between annotators at three levels of justice. We calculated Fleiss’ Kappa values for each document annotated by the three annotators, using scale interpretations to define agreement categories. At the first level, most documents had Fleiss’ Kappa values ​​in the Good category, with an average agreement of 0.746. Certain documents, such as Document Id 1, reached the Very Good category with a value of 0.819. At the appeal level, there was variation in the level of agreement, with an average Fleiss’ Kappa of 0.671. Some documents, such as Document Id 3629, had a Very Good value (0.813), while others, such as 3628 and 3676, were in the Moderate category. Most documents were in the Good category at the cassation level, with an average Fleiss’ Kappa value of 0.698. Document Id 3819 achieved the highest value in the Very Good category (0.815). Overall, the inter-annotator agreement on the dataset shows good consistency, with most documents falling in the Good category, reflecting high annotation quality.

**Table 5:** Results of calculation of agreement between annotators.

Court Level	Document Id	Fleiss’ Kappa	Agreement Category
First Instance	1	0.8192803925	Excellent
	2	0.6845484061	Good
	3	0.7833902162	Good
	4	0.6822107081	Good
	1209	0.7985781991	Good
	1210	0.7803784861	Good
	1211	0.7282813481	Good
	2417	0.6577946768	Good
	2418	0.7929292929	Good
	2419	0.7362440191	Good
Average	–	0.7463635745	Good
Appeallate	3627	0.6548399842	Good
	3628	0.520284276	Moderate
	3629	0.813999021	Excellent
	3676	0.4396399293	Moderate
	3677	0.7087378641	Good
	3678	0.657148569	Good
	3679	0.7939684937	Good
	3725	0.6905979106	Good
	3726	0.7464433639	Good
	3727	0.6845952024	Good
Average	–	0.6710254614	Good
Cassation	3774	0.6739587716	Good
	3775	0.7343209081	Good
	3776	0.7356567483	Good
	3795	0.5905552187	Moderate
	3796	0.7366354193	Good
	3797	0.6420192308	Good
	3816	0.674550004	Good
	3817	0.7319470392	Good
	3818	0.6470308034	Good
	3819	0.8151966399	Excellent
Average	–	0.6981870783	Good

The manual annotation process in the current study resulted in the creation of the IDTheftCase-JudgmentCorpus [9], a rigorously structured dataset rich in legal information. The corpus contains 2687 documents relating to court judgments of three levels of the judiciary, thereby providing a representative and valuable tool to support research in NER, legal text analysis, and large-scale applications in NLP. The annotation quality was maintained through a rigorous process and was then verified through Fleiss’ Kappa, which found high inter-annotator agreement. This dataset greatly expands available resources for legal natural language processing in the Indonesian context. It presents new opportunities to explore and harvest judicial documents for higher-order legal systems systematically.

Table 6 presents comprehensive statistics about the annotated dataset across the three levels of the judiciary. These statistics point to the richness and complexity of the dataset, showing its usefulness for NLP model development and empirical research in law. The structured dataset allows for insightful analysis of legal entity distribution, case metadata, and linguistic patterns across judicial hierarchies, forming a solid backbone for subsequent computational law research.

**Table 6:** The statistics of the CSV file dataset.

No	Item	First Instance	Appellate	Cassation
1	#decision documents	2478	147	62
2	#tokens	15,816,311	600,847	151,069
3	#unique tokens	112,992	19,808	6919
4	Avg count of tokens per document	6382.692	4087.395	2436.597
5	#annotated entities	107,402	6569	2847
6	#tagged tokens (“B” and “I” tags)	841,159	75,015	54,830
7	#untagged tokens	14,975,152	525,832	96,239

These statistics add value to the dataset as a fertile legal natural language processing research source. By offering rich distributions at various judicial levels, the dataset allows for greater insight into legal entity occurrence in Indonesian court verdicts while supporting the development and testing of machine learning models in scenarios with limited legal resources.

In addition, the methodological structure presented in this work includes structured annotation procedures, entity taxonomies, and validation through inter-annotator agreement. This combination offers a reusable and scalable approach for building high-quality legal corpora. This process facilitates the extraction of fine-grained features that are essential for downstream NLP tasks, including legal document classification, information retrieval, and indictment prediction. As such, the method serves not only as a data preparation protocol but also as a strategic enabler in advancing the development of Legal Artificial Intelligence within judicial systems. Furthermore, its adaptability to various linguistic and legal contexts provides a strong foundation for cross-jurisdictional applications and contributes to the integration of deep learning techniques in real-world legal environments.

## Limitations

While effective at producing formatted legal data from Indonesian court decisions, the proposed annotation method is not invulnerable to limitations. First, the method relies on the availability of complete and publicly accessible legal documents. In most cases—most significantly at the cassation and appellate levels—complete court decisions either do not exist or are not complete, limiting the number of documents that can be processed using this method.

Second, the annotation is entirely manual; while this results in a high-quality output, it is necessarily time-consuming and resource-intensive. For instance, of 3479 first-instance decisions that were gathered and marked as appropriate for annotation, it was only possible to process 2478 due to time and available personnel constraints. This situation poses a genuine limitation in the scalability of the method unless semi-automated or active learning techniques are integrated.

Thirdly, the approach is now being used for a particular category of criminal cases (theft), which can lead to thematic bias. Finally, since the availability of appellate cases determines the choice of first-instance cases, some institutional or geographical bias could exist.

## Ethics statements

This research did not entail human subjects, conducting animal experiments, or utilizing social media source data. All the data utilized in this study were gathered from publicly accessible court verdict documents published on the official website of the Supreme Court of the Republic of Indonesia (https://putusan3.mahkamahagung.go.id/). The previous documents are in the public domain, and no personal or confidential information that needs informed consent was utilized.

## CRediT authorship contribution statement

**Eka Qadri Nuranti:** Conceptualization, Methodology, Data curation, Writing – original draft, Supervision. **Naili Suri Intizhami:** Project administration, Formal analysis, Validation, Writing – review & editing. **Evi Yulianti:** Writing – review & editing, Visualization. **A. Muh. Iqbal Latief:** Data curation, Validation, Investigation. **Osama Iyad Al Ghozy:** Data curation, Resources.

## Declaration of competing interest

The authors declare that they have no known competing financial interests or personal relationships that could have appeared to influence the work reported in this paper.

## Data Availability

The annotated data supporting the findings of this study are publicly available in Mendeley Data at. https://doi.org/10.17632/48x9xm7rkf.3
